# NO-ferroheme is a signaling entity in the vasculature

**DOI:** 10.1038/s41589-023-01411-5

**Published:** 2023-09-14

**Authors:** Andrei L. Kleschyov, Zhengbing Zhuge, Tomas A. Schiffer, Drielle D. Guimarães, Gensheng Zhang, Marcelo F. Montenegro, Angela Tesse, Eddie Weitzberg, Mattias Carlström, Jon O. Lundberg

**Affiliations:** 1 Department of Physiology and Pharmacology, Biomedicum, Karolinska Institutet, Solna, Sweden; 2 Freiberg Instruments GmbH, Freiberg, Germany; 3 National Clinical Research Center for Child Health, National Children’s Regional Medical Center, The Children’s Hospital, Zhejiang University School of Medicine, Hangzhou, China; 4 Department of Molecular Biosciences, the Wenner-Gren Institute, Stockholm University, Stockholm, Sweden; 5 Nantes Université, INSERM, CNRS, UMR1087, l’Institut du Thorax, Nantes, France

**Keywords:** Cell signalling, Pharmacology, Mechanism of action

## Abstract

Despite wide appreciation of the biological role of nitric oxide (NO) synthase (NOS) signaling, questions remain about the chemical nature of NOS-derived bioactivity. Here we show that NO-like bioactivity can be efficiently transduced by mobile NO-ferroheme species, which can transfer between proteins, partition into a hydrophobic phase and directly activate the sGC–cGMP–PKG pathway without intermediacy of free NO. The NO-ferroheme species (with or without a protein carrier) efficiently relax isolated blood vessels and induce hypotension in rodents, which is greatly potentiated after the blockade of NOS activity. While free NO-induced relaxations are abolished by an NO scavenger and in the presence of red blood cells or blood plasma, a model compound, NO-ferroheme-myoglobin preserves its vasoactivity suggesting the physiological relevance of NO-ferroheme species. We conclude that NO-ferroheme behaves as a signaling entity in the vasculature.

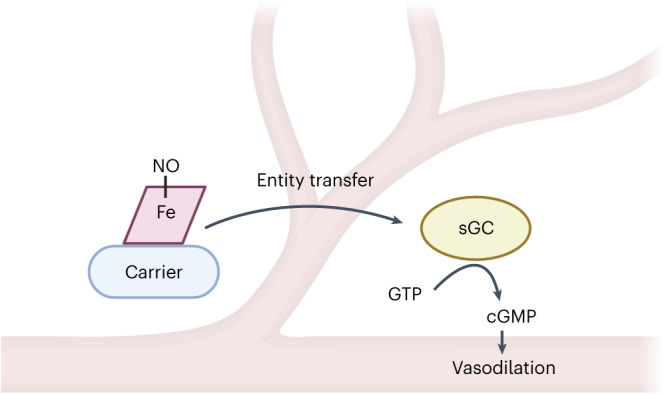

## Abstract (web summary)

Questions remain on the nature of the bioactivity of nitric oxide (NO) synthase signaling despite its wide appreciation. Here the authors describe NO-ferroheme as a vascular signaling species, whose biological activity is unrelated to the release of free nitric oxide, but allows it to travel protected to its main target guanylyl cyclase.

## Main

Nitric oxide (NO) synthase (NOS)-mediated signaling is essential for the function of cardiovascular, nervous, renal and immune systems and so on^1–3^. Canonically, NOS catalyzes the conversion of l-arginine and molecular oxygen to NO, which then diffuses within and between cells and binds to the prosthetic ferroheme group of soluble guanylyl cyclase (sGC) leading to rapid and strong enzyme activation.

Despite wide appreciation and decades of research efforts, several fundamental and methodological aspects of NOS signaling remain unsolved^2,4^. The concept of freely diffusing NO as the major NOS-derived effector in cellular signaling may be questioned. In both blood and tissues, the presence of high concentrations of ferroheme proteins, such as hemoglobin (Hb) and myoglobin (Mb), with potent NO scavenging properties, should theoretically impede free NO signaling. Indeed, although authentic NO in solution can be accurately measured in the pM to low nM range, it has still proven nearly impossible to measure its biological levels. The question of free NO signaling has also been raised in relation to vasodilation by nitroglycerin (NTG), where a discrepancy between vasoactivity and NO release has been observed^5,6^.

Canonically, the activation of sGC by NO requires a mature heme-containing heterodimer. However, studies indicate that as much as 40–80% of the total cellular sGC pool consists of heme-free (apo)-sGC^7^. This raises the question as to why cells and tissues maintain such high levels of NO-insensitive apo-sGC and that there might be other means, apart from free NO, to activate this enzyme. In somewhat overlooked studies in the early 1980s, different groups could show that various nitrosylated hemeproteins could activate both heme-free and mature sGC in vitro^8–11^. Even after the discovery of NO as the endothelium-derived relaxing factor (EDRF)^12,13^, doubts have been raised on the sole role of free NO in NOS-mediated signaling, and it was argued that EDRF is not free NO but rather a mixture of NO group-containing compounds^14–17^. Despite these findings, nitrosylated heme has been discussed mostly in relation to NO scavenging and thereby as an intravascular surrogate measure of NO generation^18^. In addition, red blood cell (RBC) nitrosyl heme has been proposed as an intermediate precursor involved in the export of NO-like bioactivity^19–22^. Indeed, after more than 30 years of intensive research efforts, the intrinsic contradictions and challenges associated with the NO-gas signaling dogma have not been overcome and the search for alternative NO-related mediators accounting for NOS signaling is still relevant^2^.

Recently, Kleschyov put forward NO-ferroheme signaling hypothesis—it was argued that mobile NO-ferroheme species may serve as efficient and potentially advantageous NOS-derived signaling molecules^4^. This theory is supported by an early and recent experimental work of Stuehr et al. who showed that low doses of NO can cause heterodimerization of the sGCα1β1 subunits by causing heme insertion into the sGCβ1 subunit^23,24^. In addition, stimulation of NOS with calcium ionophore A23187 in cell coculture experiments mobilizes intracellular heme for insertion into the apo-sGCβ subunits, leading to the formation of a functional sGC heterodimer in reporter cells^25^. Together, all the above suggest that mobile/protein exchangeable NO-ferroheme species could account for many of the NOS-dependent physiological effects, but details are still to be dissected.

In this study, we explore the hypothesis that NO-ferroheme is a signaling entity in its own right in the cardiovascular system. By synthesizing several different paramagnetic NO-ferroheme preparations, we have examined their bioactivity and downstream signaling pathway. The biological responses to protein-bound or protein-free NO-ferroheme species are compared to those of NO to allow insight into signaling mechanisms.

## Results

### Different NO-ferroheme preparations relax blood vessels

**Fig. 1:**
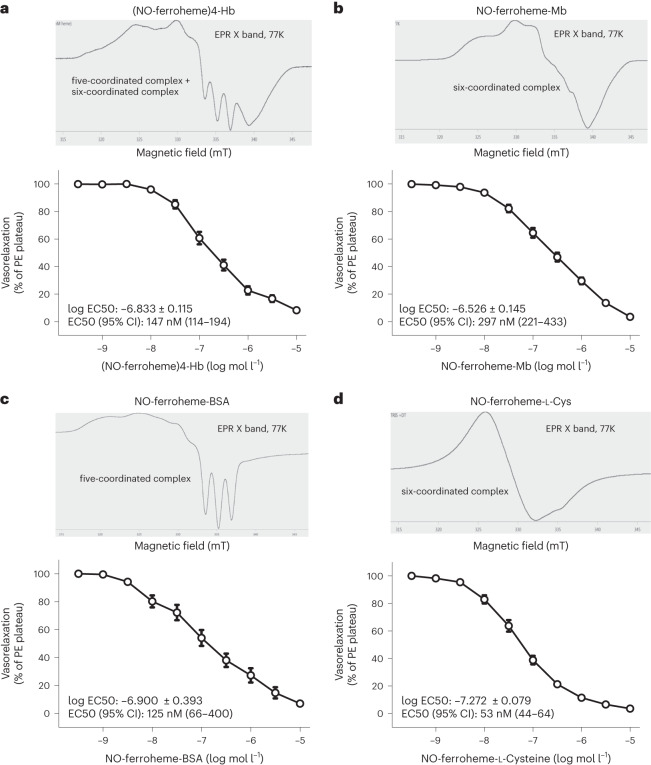
Different NO-ferroheme preparations potently relax blood vessels. **a**–**d**, EPR spectra (X band; 77K) of different NO-ferroheme preparations together with ex vivo vasorelaxation responses of aortic rings to increasing concentrations of (NO-ferroheme)4-Hb (*n* = 14) (**a**), NO-ferroheme-Mb (*n* = 31) (**b**), NO-ferroheme-BSA (*n* = 4) (**c**) and NO-ferroheme-l-Cys (*n* = 27) (**d**), using the myograph system in the presence of 0.1 mM L-NAME. *n* represents the number of mouse aortic rings used in different myograph chambers. Vasorelaxation responses are shown as percent of PE-induced plateau, and the data are presented as mean ± s.e.m. Half maximal EC50 values were calculated using least squares nonlinear regression analysis and are presented as log and absolute values with a 95% CI. CI, confidence interval; EC50, effective concentration; PE, phenylephrine. Source data

It is well known that authentic NO is a potent vasorelaxant. It is generally assumed that NO diffuses within the vascular wall to react with intracellular ferroheme-sGC, resulting in the formation of a ternary NO-ferroheme-sGC complex, activation of the sGC/PKG pathway and subsequent vasorelaxation. However, as it has been shown recently, sGC largely exists in the heme-free form (apo-sGC), which does not respond to NO^7^. We, therefore, asked whether exogenously supplied NO-ferroheme can reach and activate vascular sGC to promote relaxations. To this end, we synthesized several different NO-ferroheme preparations, characterized them with electron paramagnetic resonance (EPR) and tested them for vasoactivity. We find that both six-coordinated complexes, including (NO-ferroheme)4-Hb, NO-ferroheme-Mb and NO-ferroheme-l-cysteine (Cys), and five-coordinated complexes (NO-ferroheme-BSA) effectively relax mouse aorta in the mid nM range (Fig. 1a–d). NO-ferroheme-l-Cys was more potent compared to NO-ferroheme-Mb (Supplementary Fig. 1). In aggregate, these results suggest that the active mediator in all these different preparations is a mobilized NO-ferroheme entity. Any possible role of S-nitrosothiols, which potentially might be formed during the synthesis, can be excluded as the equine muscle Mb used in these studies lacks both disulfides and sulfhydryl groups^26^. Next, we compared NO-ferroheme-Mb relaxations in rings with or without a functional endothelium. Somewhat unexpectedly, we found that removal of the endothelium slightly but significantly attenuated the NO-ferroheme-Mb relaxations, although this occurred only at the highest doses (Supplementary Fig. 2a). This was different from the NO responses for which the relaxation curves were identical (Supplementary Fig. 2b). These results suggest that the endothelium does not represent a major barrier for diffusion of the NO-ferroheme entity to vascular smooth muscle sGC. The slight right shift of the relaxation curve at the higher concentrations of NO-ferroheme-Mb used indicates that this entity may also additionally target some yet unknown signal transduction pathway/structure within the endothelium to promote efficient vascular smooth muscle relaxation.

### NO-ferroheme vasorelaxation is mediated by sGC/PKG

**Fig. 2:**
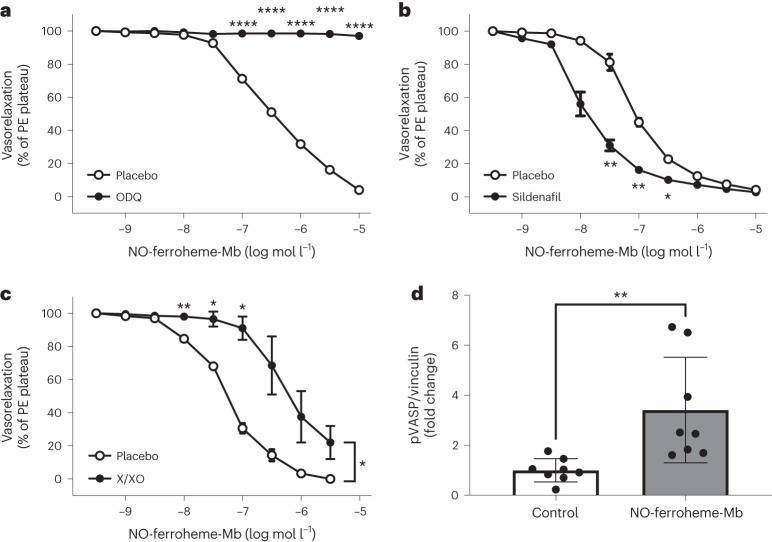
NO-ferroheme vasorelaxation is mediated by the sGC/PKG pathway. Ex vivo vasorelaxation responses of mouse aortic rings to increasing concentrations of NO-ferroheme-Mb. **a**,**b**, The responses to NO-ferroheme-Mb were abolished by simultaneous treatment with ODQ (10 μM), a cell-permeable and potent inhibitor of sGC (placebo, *n* = 4; ODQ, *n* = 4) (**a**), but sensitized by the PDE5 inhibitor, sildenafil (1 μM) placebo, *n* = 4; sildenafil, *n* = 4) (**b**). **c**, Simultaneous addition of X (0.37 mM) and XO (5 mU ml^−1^), to induce superoxide production and alter the redox status, attenuated the response to NO-ferroheme-Mb (placebo, *n* = 3; X/XO, *n* = 2). **d**, In isolated rat aortic segments treated with the NOS inhibitor, L-NAME (300 µM), co-incubation with NO-ferroheme-Mb (0.5 µM) significantly increased pVASP, thus indicating the activation of the sGC/PKG pathway. Data in **a**–**c** were analyzed by two-way repeated measures ANOVA followed by Šídák’s multiple comparisons test. Vasorelaxation responses are shown as percent of PE-induced plateau, and the data are presented as mean ± s.e.m. *n* in **a**–**c** represents the number of mouse aortic rings used in different myograph chambers. Data in **d** were analyzed by nonparametric Kruskal–Wallis test followed by Dunn’s multiple comparisons test and presented as mean ± s.d. Dots in **d** represent different aortic segments from three different rounds of western blot analysis. In **a**–**d**, *P ≤ 0.05, **P ≤ 0.01 and ****P ≤ 0.0001. Source data

Next, we asked whether NO-ferroheme promotes relaxation via activation of vascular sGC. The NO-ferroheme-Mb-induced relaxations were effectively blocked by the sGC inhibitor, 1H-[1,2,4]Oxadiazolo[4,3-a]quinoxalin-1-one (ODQ) (Fig. 2a), and potentiated by the phosphodiesterase type 5 (PDE5) inhibitor, sildenafil (Fig. 2b) clearly suggesting an sGC–cGMP-dependent effect. To study the potential sensitivity of NO-ferroheme-mediated relaxations to oxidative stress, we performed relaxation studies in the presence of the superoxide-generating system, xanthine (X)/xanthine oxidase (XO). We observed attenuated vasorelaxant effects of NO-ferroheme-Mb (Fig. 2c), pointing to the similarity of NO-ferroheme species with EDRF originally described in ref. ^27^. We next asked whether NO-ferroheme species increase the sGC/PKG-dependent phosphorylation of vasodilator-stimulated phosphoprotein at Ser239 (pVASP)^28^. We found that exposure of rat aortic rings to NO-ferroheme-Mb significantly increased the vascular levels of pVASP (Fig. 2d), which suggests PKG activation, thereby suggesting NO-ferroheme signals via this pathway. Taken together, this set of experiments suggests that NO-ferroheme-Mb-induced vasorelaxation is due to the activation of the sGC/PKG pathway.

### NO-ferroheme bioactivity is unrelated to NO release

**Fig. 3:**
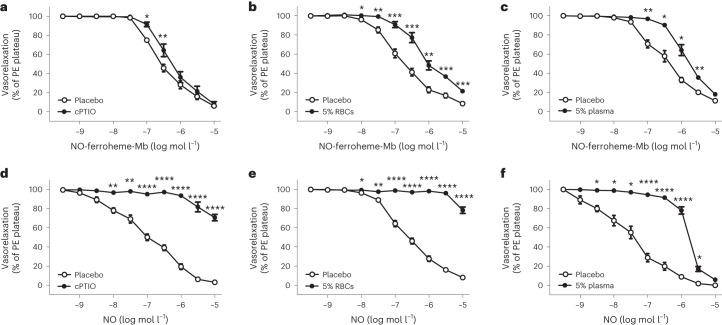
The bioactivity of NO-ferroheme is unrelated to the release of NO. **a**–**f**, Ex vivo vasorelaxation responses of mouse aortic rings to increasing concentrations of NO-ferroheme-Mb (**a**–**c**) and NO (**d**–**f**), in the absence or presence of the NO scavenger cPTIO (100 μM) (**a**,**d**), RBC (5 vol%) (**b**,**e**) or blood plasma (5 vol%) (**c**,**f**). The vasodilatation by NO was effectively blocked or greatly attenuated by cPTIO (**d**), RBC (**e**) or plasma (**f**). In sharp contrast, none of these scavengers had any major effect on NO-ferroheme-Mb-induced vasorelaxations (**a**–**c**). Data in **a**–**f** were analyzed by two-way repeated measures ANOVA followed by Šídák’s multiple comparisons test. Vasorelaxation responses are shown as percent of PE-induced plateau, and the data are presented as mean ± s.e.m. The number (*n*) of mouse aortic rings used in different myograph experiments were as follows: **a**, placebo, *n* = 5; cPTIO, *n* = 6; **b**, placebo, *n* = 14; RBC, *n* = 14; **c**, placebo, *n* = 6; plasma, *n* = 6; **d**, placebo, *n* = 7; cPTIO, *n* = 8; **e**, placebo, *n* = 27; RBC, *n* = 16; and **f**, placebo, *n* = 6; plasma, *n* = 6. *P ≤ 0.05, **P ≤ 0.01, ***P ≤ 0.001 and ****P ≤ 0.0001. Source data

To confirm the idea of NO-ferroheme-mediated relaxations being due to the entire complex rather than NO release, we performed vessel relaxation experiments in the presence and absence of the known NO scavenger, cPTIO. While cPTIO only marginally affected the NO-ferroheme-Mb relaxations (Fig. 3a), it almost abolished the relaxations by authentic NO (Fig. 3d). Next, we thought to compare the biological relevance of NO-ferroheme species and NO, performing the relaxation studies in the presence of intact RBCs. Because NO is a freely diffusible small molecule, it should be effectively scavenged by the intra-erythrocytic oxy-Hb. The addition of 5 vol% of RBCs nearly completely prevented the NO relaxations, while, in contrast, the NO-ferroheme-Mb relaxations were largely preserved (Fig. 3b,e). Similar results were obtained when 5 vol% of blood plasma was added to organ chambers (Fig. 3c,f). These results indicate once more a limitation of a free NO signaling function and favor the NO-ferroheme-carrier perspective.

**Fig. 4:**
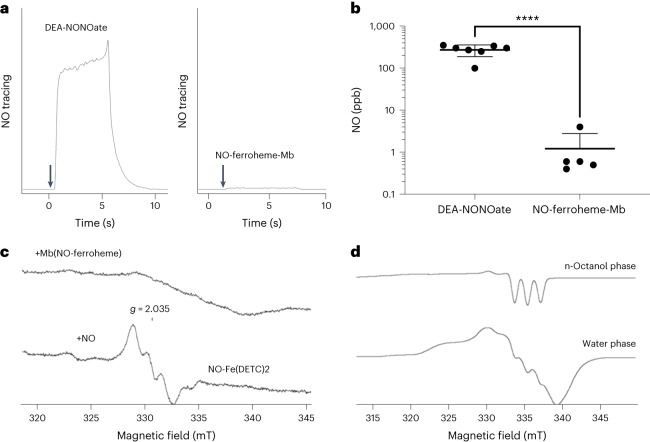
No release of NO from NO-ferroheme-Mb. **a**,**b**, Original tracings (**a**) and mean NO concentrations (**b**) in headspace gas of solutions containing mouse aortic rings and 1 µM of an NO donor (DEA-NONOate, *n* = 7) or NO-ferroheme-Mb (Mb-NO, *n* = 5). **c**, EPR spectra of rat aortic rings incubated either with 10 µM NO-ferroheme-Mb (upper trace) or with 10 µM spermine NONOate (lower trace) in the presence of hydrophobic NO spin trap, colloid Fe(DETC)2 (100 µM; 30 min), following washout. Intravascular NO was detected only in NO donor-treated rings; in NO-ferroheme-Mb-treated rings, a six-coordinated NO-ferroheme EPR signal can be observed. Representative EPR spectra of three independent experiments. **d**, Partition of NO-ferroheme entity as five-coordinated species from NO-ferroheme-Mb water solution to 1-octanol phase. NO-ferroheme-Mb solution (1 mM; 1 ml) was mixed with an equal volume of deoxygenated 1-octanol, vortexed for 5 min and centrifugated (5 min). Samples from the upper (1-octanol) phase and lower (water) phase were analyzed by EPR at 77K. The 1-octanol samples exhibited an EPR signal characteristic of five-coordinated NO-ferroheme species, while the water samples showed a signal of parent six-coordinated NO-ferroheme-Mb. Double integration of the EPR signals indicated that 20–30% of NO-ferroheme groups were partitioned from Mb to 1-octanol phase. Representative spectra of two experiments. Data presented in **b** were analyzed by unpaired *t*-test and presented as mean ± s.d. *P* value for statistically significant difference is indicated in **b**. Source data

Next, we attempted to detect NO release from NO-ferroheme-Mb using a headspace chemiluminescent assay. In these experiments, we incubated mouse aortic rings in oxygenated buffer in the presence of NO-ferroheme-Mb or an NO donor (DEA-NONOate) at equimolar and equipotent concentrations and used headspace for NO measurements. While DEA-NONOate generated robust NO signals in the headspace gas, only minute amounts were released from NO-ferroheme-Mb preparations (Fig. 4a,b). Similar results were obtained using the intravascular EPR spin trapping technique with a hydrophobic NO trap, colloid Fe(DETC)2. With authentic NO, a clear intravascular NO-Fe(DETC)2 signal was noted with EPR, while NO-ferroheme-Mb gave no such signal (Fig. 4c).

Next, using a water/1-octanol approach and EPR, we demonstrate the partition of a five-coordinated paramagnetic NO-ferroheme entity from the water-soluble six-coordinated NO-ferroheme-Mb to the hydrophobic 1-octanol phase (20–30% during 15 min; Fig. 4d). When we made a similar experiment with NO-ferroheme-l-Cys, the partition to 1-octanol was nearly complete and accomplished much faster (1–2 min; Supplementary Fig. 3). We next tested whether exogenous NO-ferroheme species can partition to intact cells. To this end, NO-ferroheme-Mb or NO-ferroheme-l-Cys were added to a HEK293A cell suspension, and then the cell fractions were studied by EPR. We found that both preparations can donate NO-ferroheme to cells (Supplementary Fig. 4a,b). Again, the partition of NO-ferroheme to cells was faster and more complete in the case of NO-ferroheme-l-Cys. When we injected our NO-ferroheme-Mb preparation into anesthetized rats (i.v.; 1600 nmol kg^−1^) and tested blood samples 20–30 min later, we found that the NO-ferroheme signal was present in RBC only but was absent in plasma samples (Supplementary Fig. 5). These results again demonstrate that NO-ferroheme is mobile and can partition from a host protein to RBCs and tissues.

**Fig. 5:**
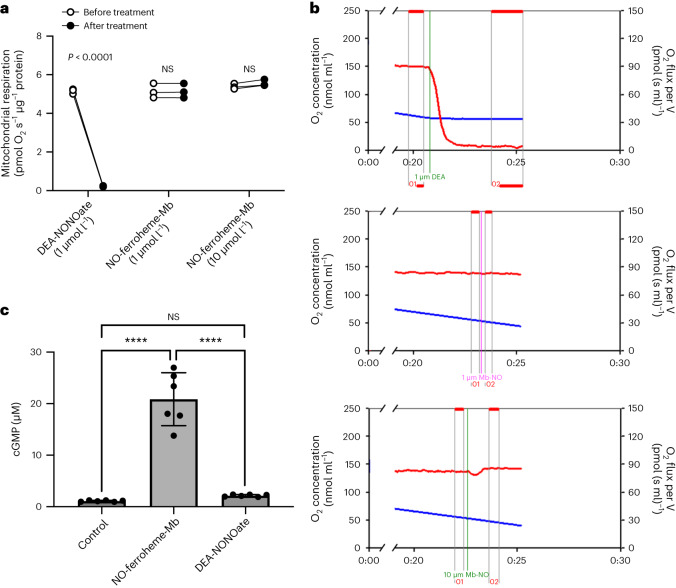
Effects of NO-ferroheme on mitochondria and apo-sGC activation. **a**,**b**, The effect of NO-ferroheme-Mb and the NO donor DEA-NONOate on mitochondrial (CI + CII) state 3 respiration evaluated by high-resolution respirometry. **a**, DEA-NONOate clearly inhibited mitochondrial respiration, whereas NO-ferroheme-Mb had no inhibitory effect. **b**, Original tracings showing mitochondrial respiration in an oxygraph and the effects of DEA-NONOate (1 μM, top) and Mb-NO (1 μM, middle and 10 μM, bottom). DEA-NONOate completely inhibited (CI + CII-dependent) state 3 respiration (red curve, top), whereas NO-ferroheme-Mb had no effect (red curve, middle and bottom). Blue curves represent oxygen concentration in the chamber. **c**, Apo-sGC was prepared and supplemented with either NO-ferroheme-Mb (10 μM) or DEA-NONOate (10 μM), followed by an analysis of cGMP production using an ELISA kit. Apo-sGC was clearly activated by NO-ferroheme-Mb, but not by NO released from DEA-NONOate. Respirometry data in **a** were repeated three times and analyzed by paired two-way repeated measures ANOVA followed by Šídák’s multiple comparisons test. Data in **c** were analyzed by nonparametric Kruskal–Wallis test followed by Dunn’s multiple comparisons test. Dots connected with lines in **a** represent paired data from three independent experiments. Dots in **c** represent the number of independent observations, and the data are presented as mean ± s.d. **a**,**c**, *P* values for statistically significant differences are indicated. ****P ≤ 0.0001. Differences not significant are indicated as NS. Source data

In a different functional model, we used high-resolution respirometry to study respiration in isolated mitochondria. It is well known that NO inhibits respiration through competition with oxygen at cytochrome c oxidase^29^, and this inhibitory effect of NO was clearly seen here (Fig. 5a,b). Strikingly, with the same or even higher concentrations of NO-ferroheme-Mb, no signs of inhibition were noted (Fig. 5a,b). Altogether, these results clearly demonstrate that NO-ferroheme species are not acting via the release of free NO. Rather these suggest that NO-ferroheme behaves as a functional signaling entity in its own right.

### NO-ferroheme but not NO activates apo-sGC

Studies done before the discovery of NO as a signaling molecule in mammals had shown that various preparations of heme-NO can activate heme-deficient sGC^8–11^. In light of the data generated here, we decided to try to reproduce parts of those old experiments. Apo-sGC was prepared as described^30^, and we then exposed this enzyme to NO-ferroheme-Mb or DEA-NONOate and measured cGMP generation. The results clearly show the activation of apo-sGC by NO-ferroheme-Mb but not by NO (Fig. 5c). This suggests that NO-ferroheme could be a signaling entity that directly incorporates into apo-sGC to activate it.

### NO-ferroheme species potently reduce blood pressure

**Fig. 6:**
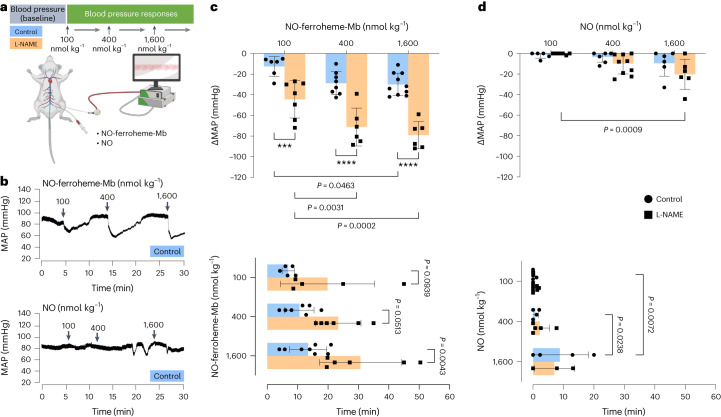
Cardiovascular effects in vivo of NO-ferroheme-Mb and NO. **a**, Blood responses to intravenous injection of different doses of NO-ferroheme-Mb and authentic NO were analyzed in anesthetized Wistar rats with regular water or supplemented with L-NAME (1 g l^−1^). **b**, Traces of blood pressure in response to increasing bolus doses of NO-ferroheme-Mb and authentic NO in control rats. Administration of NO-ferroheme-Mb, but not authentic NO, showed profound and dose-dependent reductions of blood pressure, which were potentiated in rats with NOS inhibition. **c**,**d**, Summarized blood pressure responses and the duration of the responses to different doses of NO-ferroheme-Mb (**c**) and authentic NO (**d**) in control and L-NAME-treated rats. Data were analyzed with two-way ANOVA (mixed-effects model) followed by Šídák’s multiple comparisons test. The dots in **c** and **d** (circles, controls; squares, L-NAME) represent the responses in different animals, and data are presented in bars as mean ± s.d. In **c**,**d**, *P* values for statistically significant differences are indicated. Blue shading denotes control and orange shading denotes L-NAME. ***P ≤ 0.001 and ****P ≤ 0.0001 between the same dose given in control and L-NAME-treated rats. Source data

Having observed the potent vasorelaxant effect of NO-ferroheme complexes in vitro, we next wanted to investigate if they were bioactive in vivo as well. To this end, increasing bolus doses of authentic NO, NO-ferroheme-Mb or NO-ferroheme-l-Cys were given intravenously to controls or L-NAME pretreated anesthetized rats, and blood pressure responses were monitored (Fig. 6a). Authentic NO significantly reduced blood pressure but only at the highest dose (1600 nmol kg^−1^), and this effect was not affected by L-NAME (Fig. 6b,d and Supplementary Fig. 6d). In contrast, administration of NO-ferroheme-Mb demonstrated a potent dose-dependent blood pressure lowering effect, which was greatly potentiated in rats pretreated with L-NAME (Fig. 6b,c and Supplementary Fig. 6c). Injection of NO-ferroheme-l-Cys also elicited a dose-dependent and L-NAME-enhanced hypotensive effect, albeit shorter in duration when compared to NO-ferroheme-Mb (Supplementary Fig. 7). When we compared the integral response (AUC) induced by all three compounds, we found that the activity of NO-ferroheme-Mb was higher than that of NO-ferroheme-l-Cys, which in turn was more potent than authentic NO (Supplementary Fig. 8). In control experiments, neither ferro-Mb (Mb^2+^) nor ferri-Mb (Mb^3+^) had any effect on blood pressure (Supplementary Fig. 6b).

These data demonstrate that NO-ferroheme species are potent hypotensive agents, demonstrating enhanced activity in protein-bound form and after inhibition of NOS, with potency and duration greatly exceeding that of free NO.

## Discussion

Here we show that NO-ferroheme (naked and protein-bound) activates the sGC–cGMP–PKG pathway, promotes vasorelaxation and reduces blood pressure working as a signaling entity without intermediacy of free NO. In contrast to free NO, the NO-ferroheme species can preserve its vasoactivity in the presence of blood and other NO scavengers, can be carried by various proteins, transfer between proteins and partition into cellular membranes. These chemical and biological properties of NO-ferroheme may be advantageous for a paracrine and endocrine signaling function.

NOS is known to perform surprisingly diverse physiological functions, and the role of NO as the sole NOS-derived effector has been questioned^2,4^. The chemical identification of NOS-derived bioactivity in intact cells and tissues is a challenging task of modern biochemistry and a subject of intense debate. As an example, it has been notoriously difficult to unequivocally detect NO being released both from purified NOS and from intact cells, despite available sensitive methodologies. Several NO-related compounds have been investigated as potential mediators of NOS bioactivity, including S-nitrosothiols^31,32^, peroxynitrite^33,34^, nitrosopersulfide^35,36^ and dinitrosyl-iron complexes^37,38^. Recently, protein-exchangeable NO-ferroheme species have been proposed to account for many puzzling aspects of NOS signaling^4^, although direct evidence of signaling by this species has been lacking. Indeed, the final product of NOS catalysis is in fact NO-heme^39^, and at the same time, the activation state of sGC is also linked to the NO-heme-sGC formation. One may, therefore, ask the following simple question: why would nature choose to first decompose an already optimal sGC activator (NO-heme) and then reconstruct it again at the sGC site? A possibility of a chaperon-assisted NO-ferroheme entity transfer from endothelial nitric oxide synthase (eNOS)/neuronal nitric oxide synthase (nNOS) to tissue globins or directly to sGC has been proposed previously^4^. In any case, if eNOS/nNOS releases free NO, it would likely react rapidly with the intracellular loosely bound heme pool, forming the same NO-heme. The estimated levels of this heme pool are about 100 nM^40,41^ (which is probably higher than the levels of heme-sGC), while the reaction rate of NO with free heme is comparable with that of heme-sGC^42^. In fact, the heme of sGC may be classified as a loosely bound heme (*K*_d_: 100–200 nM (ref. ^10^)).

Stuehr et al. demonstrated that low doses of NO can cause heterodimerization of the sGCα1β1 subunits via heme insertion into sGC^24^. This group also showed that low levels of exogenous NO rapidly induce NO-heme formation, its relocation and sGC activation within reporter cells^43^. Thus, NO bioactivity previously seen already at pM concentrations^44^ may be well mediated by a labile NO-ferroheme-dependent mechanism. Strong support for NO-ferroheme signaling comes from early sGC studies showing efficient transfer of NO-heme unit from nitrosylated hemoproteins to sGC, together with enzyme activation^8–11^. Moreover, Sweeny et al. recently demonstrated sGC activation in cells by heme-NO preparation, without apparent NO release^23^.

One aim of this study was to test if exposure of blood vessels to NO-ferroheme species results in vasorelaxation. To this end, we synthesized several paramagnetic NO-ferroheme preparations—(NO-ferroheme)4-Hb and NO-ferroheme-Mb—as they represent the most abundant hemoproteins in the body. In addition, we specifically selected equine muscle Mb, as it lacks sulfhydryl groups; thus, any generation of protein S-nitrosothiols in this preparation can be excluded. After being fully Fe-nitrosylated, these hemoproteins form mainly six-coordinated NO-ferroheme species. We also prepared NO-ferroheme bound to BSA, the major carrier transport protein in blood plasma, which forms five-coordinated species^45^ and thus loosely binds NO-ferroheme. Finally, we prepared the low molecular weight complex, NO-ferroheme-l-Cys, which is supposed to be six-coordinated. We found that all these paramagnetic NO-ferroheme preparations relaxed mouse aortas with comparable potencies, with globin-free NO-ferroheme-l-Cys being significantly more potent than NO-ferroheme-Mb. The comparable efficiency of five-coordinated and six-coordinated protein-bound NO-ferroheme complexes is rather surprising as migration of NO-ferroheme from a protein requires the breakage of a rather strong bond. However, the sixth position in NO-ferroheme can potentially be replaced and occupied by a wide variety of endogenous ligands, including amino acids, peptides, H_2_S and some drugs. Whether such modification of NO-ferroheme will affect its bioactivity is an interesting question for future studies.

Our functional in vitro studies with inhibition of vascular sGC and PDE5 clearly implicate an sGC-dependent mechanism of vasorelaxation. However, the intracellular loci and the relative sensitivity of different sGC isoforms to exogenous NO-ferroheme species warrant further investigation. Our data also implicate a potential role of apo-sGC and activation of PKG in the mechanism of NO-ferroheme vasodilation. To prove the rather provocative idea of NO-ferroheme bioactivity being independent of free NO, we used several different approaches and protocols, and all our findings independently support the NO-ferroheme entity mechanism. Despite this strong evidence, some questions remain. As an example, the reason(s) why conditions with NO scavenging (that is, cPTIO, RBC and plasma) slightly but still significantly attenuated the NO-ferroheme-Mb relaxations is unknown. One possibility is the presence of residual NO in our preparations, and another possibility is the partition of mobilized NO-ferroheme into RBC membranes or hydrophobic parts of plasma proteins.

Although five-coordinated NO-ferroheme species are detectable by EPR in the RBC of rodents^46^ and humans^18^, the mechanism of their formation and function is still illusive. One possibility is that in vivo, endothelium-derived NO-ferroheme entities partition and accumulate in RBCs. An intriguing further question arises as to whether NO-ferroheme species formed in RBC can slowly partition back to the vascular wall. This may be relevant to the recent demonstration of the endothelium-damaging effect of RBC obtained from eNOS knockout mice^47^, as well as for the export of vasodilatory^19–22^ and cardioprotective^48^ NO-related bioactivity by RBCs.

The blood-pressure-lowering effect of NO-ferroheme preparations and its potentiation after NOS inhibition deserves further in-depth studies. Previous studies have also shown an increased hypotensive response to NTG in rats pretreated with NOS inhibitors^49^. In contrast, in the present study, the NO response was not affected by NOS inhibition.

An outstanding question is how the NO-ferroheme entity reaches the vascular sGC. Does it go through the endothelial cell (EC) membrane, transverse the EC and then pass through EC-vascular smooth muscle cell membranes to get to sGC? Our results suggested that this scenario is unlikely and it prompted us to generate a working hypothesis, according to which major events occur at the interface of blood and the vascular lumen. Firstly, the presence of an intact endothelium does not limit but slightly facilitates the NO-ferroheme relaxation (at least at higher concentrations). Secondly, while the globin-free NO-ferroheme-l-Cys more rapidly partitions to hydrophobic 1-octanol and cells (EPR data), its in vivo hypotensive effect is much shorter than that of NO-ferroheme-Mb. The longer duration of NO-ferroheme-Mb hypotension appears to correlate with the ability of Mb to retain NO-ferroheme from partitioning to lipids or associate with other nonspecific proteins. Thus, we hypothesize that a target for extracellular applied NO-ferroheme may be located at the cell surface. For example, this may be a caveolae-associated sGC^50,51^. This proposed mechanism, however, does not exclude intracellular NO-ferroheme signaling when the protein-associated species are generated by other mechanisms. The association of NO-ferroheme with a specific protein appears to be important for its signaling function. An important issue is the fate of NO-ferroheme signaling species. The very strong bond between NO and ferroheme^52^ seems to exclude a simple NO-dissociation mechanism. However, the reaction of NO-ferroheme with dioxygen and the subsequent formation of nitrate and ferriheme (Fe^3+^) is a possibility and might be considered in future studies. Finally, our findings may be also relevant to eNOS/α-Hb located to gap junctions^53^, as well as to other tissue globin proteins.

From a pharmacological aspect, NO-ferroheme species may resemble the recently developed drug, BAY58-2667 (cinaciguat), as both compounds can activate apo-sGC. However, a mechanistic difference between them exists. It is known that BAY58-2667 can activate sGC even in the presence of oxidants and in H105F mutant mice (mutation of sGCβ1), while endogenous eNOS/nNOS signaling is severely compromised under these conditions^54^. Despite this apparent advantage of BAY58-2667 over NO-ferroheme, the latter may be more suitable for the dynamic regulation of sGC activity. BAY58-2667 may potentially substitute for chronic insufficiency of endogenous NO-ferroheme, and elaboration of sensitive assays for endogenous NO-ferroheme would be important.

In conclusion, we have found that NO-ferroheme can serve as a signaling entity in its own right. These results may help to change the view on how NOS signals within the vasculature and elsewhere.

## Methods

### Ethics statement

All experimental protocols were approved by the regional Institutional Animal Care and Use Committee in Stockholm (Dnr 17128-2021 and N139/15) and performed according to the US National Institutes of Health guidelines (NIH publication 85-23, revised 1996) and EU directive 2010/63/EU for the conduct of experiments in animals. The experimental approaches for the different in vitro, ex vivo and in vivo experiments are described in detail below.

### Animals

We purchased conventional male Wistar rats (age: 8–10 weeks, weight: 200–250 g at the time of the experiments) and male C57BL/6 mice (age: 6–12 weeks, weight: 20–25 g at the time of the experiments) from Janvier Labs (France) and housed them at the animal facility (KM-B) at the Karolinska Institutet. Upon arrival, all animals were allowed at least 10-d acclimation before any experiment. Animals were kept in standard cages, housed in a temperature- (21 °C) and humidity-controlled room with 12-h light/12-h dark cycle and fed a standard rodent chow and water ad libitum.

### Synthesis of NO-ferroheme

Bovine Hb, equine skeletal muscle Mb, BSA and hemin were from Sigma Aldrich. NO gas (Linde AG, Sweden; 99.5%) was purified by passing through the column filled with granules of NaOH. To prepare (NO-ferroheme)4-Hb, solution of bovine Hb (10 ml PBS; 1 mmol l^−1^ in heme; pH 7.4; in a Thunberg tube) was first deoxygenated in a vacuum/N_2_ gas system for 15 min, then reduced with sodium dithionite (1.5 mmol l^−1^), following another 5 min deoxygenation, then treated under slow agitation with pure NO gas (1 atm, 5 min) and further evacuated (5 min) to remove dissolved but unbound NO. EPR spectra of (NO-ferroheme)4-Hb were generally similar to previously reported^55^, and we observed the formation of both six-coordinated and five-coordinated NO-ferroheme species. We ascribe this preparation as being (NO-ferroheme)4-Hb. However, we have no evidence that the tetrameric structure of Hb is preserved after complete nitrosylation of its heme groups. Therefore, it cannot be excluded that our preparation represents a mixture of α-Hb-NO and β-Hb-NO subunits. NO-ferroheme-Mb was prepared using a similar protocol; in some preparations, sodium borohydride (1.5 mmol l^−1^) was used as a reducing agent instead of sodium dithionite; EPR spectra of both these NO-ferroheme-Mb preparations were identical to those described previously^56^ (pH values of the solutions were about 6). EPR analysis showed that >95% of the protein-bound heme groups were nitrosylated. NO-ferroheme-BSA (1:1) was prepared in TRIS buffer (pH 10) using an analogous protocol; the EPR spectrum of NO-ferroheme-BSA was identical to that described previously^45^. NO-ferroheme-l-Cys (1:1; 1 mmol l^−1^) was prepared using Thunberg tubes in the following way. Hemin/DMSO solution (0.2 ml; 50 mmol l^−1^) was added to 11 ml TRIS buffer (0.1 mol l^−1^; pH 10), deoxygenated in a vacuum/N_2_ gas system for 15 min, reduced with sodium dithionite (1.5 mmol l^−1^), further deoxygenated/degassed for 5 min, treated with pure NO gas (1 atm, 5 min), evacuated to remove unbound NO (5 min) and mixed with deoxygenated solution of l-Cys (0.1 ml; 100 mmol l^−1^; pH 8). In this preparation, it is assumed that l-Cys is liganded to NO-ferroheme species. However, no direct proof of this has been obtained. The EPR spectrum at 77K indicates randomization of the structure around the heme-NO moiety, which is likely mainly six-coordinated (Fig. 1d). When NO-ferroheme-l-Cys is mixed with deoxygenated 1-octanol, it rapidly partitions to the upper phase with the quantitative transition to five-coordinated NO-ferroheme species (Supplementary Fig. 3). All NO-ferroheme preparations were divided in aliquots and immediately frozen and kept in liquid nitrogen until used. For organ chamber studies, serial dilutions of the NO-ferroheme preparations were made in deoxygenated water (N_2_; 1 h) just before the experiment.

### Preparation of authentic NO solution

NO gas (Linde AG, Sweden; 99.5%) was purified by passing it through a column filled with granules of NaOH. The authentic NO solution was prepared in a Thunberg tube connected to a system, which allows it to switch between vacuum, N_2_ gas and NO gas regimes, and equipped with a manometer. Distilled water of 10 ml was placed in the Thunberg tube, and the whole system was thoroughly deoxygenated by several vacuum/N_2_/vacuum cycles (15 min) and treated with pure NO gas at 1 atm for 15 min under continuous shaking. The headspace NO was rapidly (a few seconds) removed by vacuum and replaced with N_2_ gas. NO solution was aspirated in the N_2_-flushed 1 ml syringes and immediately frozen/stored in liquid nitrogen. Basic chemistry tells us that at 1 atm and 20 °C, water dissolves about 2 mmol l^−1^ NO. We, therefore, assume that our NO stock solution has this concentration. For organ chamber experiments, serial dilutions of the stock NO solution were made in deoxygenated water (N_2_; 1 h). This was done a few minutes before the addition to the vessels. For the in vivo studies, saline was used instead of distilled water for the preparation of the stock NO solution. We again assume that the NO concentration is about 2 mmol l^−1^. Injections (i.v.) to rats were made after thawing the stock NO solution, directly from the 1 ml syringe, where the NO solution was stored. Please note that for the NO solution used in the in vivo experiments, aliquots from the same batch did show the expected potent bioactivity in the vessel bioassay ex vivo, demonstrating that NO was not oxidized or destroyed before administration.

### Headspace chemiluminescence NO measurements

To determine if NO is released from NO-ferroheme, we used a chemiluminescence NO analyzer (Eco Physics, 77 AM). Freshly prepared solutions (5 ml, 1 µM) of NO-ferroheme-Mb or the NO donor DEA-NONOate were incubated together with four mouse aortic segments in sealed plastic syringes. A headspace of 10 ml was created in the syringe by aspirating room air, and then the syringe was immediately sealed. The sample was mixed by turning the syringe upside down repeatedly for 5 s and then left on the bench for 2 min. After this period, the headspace gas was carefully aspirated into another empty syringe and then immediately injected into the rapid response chemiluminescence detector. The NO signal was recorded using the software eDAQ (Powerchrom v.2.7.9), and peak levels of NO were determined. Ambient NO levels (<3 ppb in all cases) were subtracted from the value recorded.

### EPR studies

Spectra EPR of different NO-ferroheme preparations (1 mM in heme) were recorded using an X-band benchtop EPR spectrometer MS5000 (Magnettech-Bruker). Instrument parameters were as follows: (1) 10 mW of microwave power, (2) 0.6 mT of amplitude modulation, (3) 100 kHz of modulation frequency, (4) 60 s of sweep time and (5) 1, number of scans.

Intravascular NO levels were measured by the previously described technique using colloid Fe(DETC)2 as intracellular NO spin trap^57^. Briefly, rat aortic rings (10 mm long) were placed in a 24-well plate and incubated either with spermine NONOate (10 µM) or NO-ferroheme-Mb (10 µM) in the presence of 100 µM colloid Fe(DETC)2. After 30 min of incubation at 37 °C, aortic rings were placed in a special form and frozen in liquid nitrogen. EPR spectra were recorded at 77K using an X-band EPR spectrometer MS5000 (Magnettech-Bruker). Instrument parameters were as follows: (1) 10 mW of microwave power, (2) 0.6 mT of amplitude modulation, (3) 100 kHz of modulation frequency, (4) 60 s of sweep time and (5) 4, number of scans. The results are expressed in arbitrary units.

To test whether the NO-ferroheme entity can leave the host protein and partition to the hydrophobic phase, we mixed the NO-ferroheme-Mb solution (1 mM; 1 ml) with an equal volume of 1-octanol (has the same polarity as biological membranes), vortexed mixture for 5 min and centrifugated (5 min). Samples from the upper (1-octanol) and lower (water) phases were analyzed by EPR at 77K.

### Ex vivo vascular reactivity studies

Mice were anesthetized with isoflurane, and descending thoracic aortas were immediately isolated and dissected into 2 mm rings (*n* = 8 per mouse) in ice-cold physiological salt solution (PSS; NaCl, 130 mmol l^−1^; KCl, 4.7 mmol l^−1^; CaCl_2_, 1.6 mmol l^−1^; KH_2_PO_4_, 1.18 mmol l^−1^; MgSO_4_·7H_2_O, 1.17 mmol l^−1^; NaHCO_3_, 14.9 mmol l^−1^; glucose, 5.5 mmol l^−1^ and EDTA, 0.026 mmol l^−1^) for further vessel reactivity studies. Aortic rings were washed repeatedly with PSS to remove any RBCs and thereafter mounted onto the pins of a multiwire myograph system (Danish Myo Technology, Model 620 M). The chambers were prefilled with 8 ml PSS solution (37 °C, pH 7.4) and aerated with carbogen (95% O_2_ and 5% CO_2_). Isometric tension was recorded with PowerLab system (PowerLab 4/30). After mounting, vessels were equilibrated for 45 min. A loading force of 6 mN was added to the vessel to mimic the near-physiological pressure. After another 45 min of equilibration, vessels were contracted with KCl (70 mM) solution to determine the reactivity of the vascular smooth muscle cells (that is, viability control test). We then washed the vessels three times before performing concentration–response curves. After washing the aortic rings, we precontracted them with increasing concentrations of PE (0.1 nM to 10 μM) to reach approximately 80% of KCl-induced contraction. We excluded vessels with unstable preconstriction. A serial dilution of NO and NO-ferroheme-Mb solutions was done in deoxygenated water just before adding them to the organ bath. We performed dose–response curves to NO and different preparations of NO-ferroheme in the presence of 0.3 mmol l^−1^ L-NAME, with or without simultaneous treatment with various pharmacological compounds. Vasorelaxation responses were also compared in aortic rings with or without a functional endothelium. The endothelium was removed by gently rolling aortic rings over the microsurgery forceps. The endothelium was considered to be removed if the rings did not relax in response to acetylcholine (1 µmol l^−1^).

### Mitochondrial isolation

Kidneys were extracted from C57BL/6 mice, from which mitochondria were isolated by differential centrifugation. Tissue was homogenized in the isolation medium (250 mM sucrose, 10 mM Hepes, 1 mM EGTA, BSA 1 g l^−1^ and pH 7.4 compensated with KOH) on a slush of ice using a glass homogenizer (Potter Elvehjem) connected to a screwdriver. Homogenate was centrifuged at 700*g* for 10 min. The supernatant was collected and centrifuged at 10,000*g* for 10 min. The buffy coat on top of the pellet was removed by carefully pipetting with an isolation buffer. The pellet was resuspended in the isolation buffer and recentrifuged at 7,000*g* for 5 min followed by another washing step. The pellet was resuspended in 1 μl preservation medium per mg initial sample wet weight (5 mM EGTA, 3 mM MgCl_2_·6H_2_O, 60 mM K-lactobionate, 20 mM taurine, 10 mM KH_2_PO_4_, 20 mM HEPES, 110 mM sucrose, 20 mM histidine, 3 mM glutathione, 2 mM glutamate, 2 mM malate, 2 mM Mg-ATP, 1 g l^−1^ BSA, 20 μM vitamin E succinate and 1 μmol l^−1^ leupeptin) and left to stabilize at least 30 min before respiratory analysis.

### Effects of NO-ferroheme and NO on mitochondrial respiration

Mitochondrial respiration was evaluated by high-resolution respirometry (Oroboros, O2K) using respiration medium containing the following: (1) 0.5 mM EGTA, (2) 3 mM MgCl_2_·6 H_2_O, (3) 60 mM K-lactobionate, (4) 20 mM taurine, (5) 10 mM KH_2_P0_4_, (6) 20 mM HEPES, (7) 110 mM sucrose and (8) pH 7.1. Maximal respiratory capacity (state 3) was initiated by adding CI/CII substrates (pyruvate, 5 mM, malate 2 mM and succinate 10 mM) together with ADP (2.5 mM). NO-ferroheme-Mb (1 µM or 10 µM) or the NO donor, DEA-NONOate (1 µM) were added during state 3 respiration. Respiration was normalized to mitochondrial protein. Data were collected with Datlab, v.6.1.07. Paired student’s *t*-test was used to evaluate whether there was a statistical difference between treatments.

### Effects of NO-ferroheme and NO on cGMP production by apo-sGC

Pure human recombinant soluble guanylate cyclase was purchased from Enzo Lifesciences. Apo-sGC was prepared as previously described^30^. NO-ferroheme-Mb (10 µM) or DEA-NONOate (10 µM) was added to apo-sGC (~1 µM) dissolved in medium containing the following: (1) 10 mM GTP, (2) 10 mM HEPES, (3) 150 mM NaCl, (4) 1 mM DTT and (5) 3 mM MgCl_2_. The Eppendorf tubes were placed in a heated water bath (37 °C) for 10 min. sGC was inactivated by moving the Eppendorf tubes to a heat block (100 °C) for 3 min. The samples were stored at −80 °C until analysis. cGMP was measured using a commercial ELISA kit (Cayman Chemical; cyclic GMP ELISA kit).

### NO-ferroheme-dependent pVASP

The effect of NO-ferroheme-Mb on vascular pVASP indicative of downstream sGC activation was evaluated. Aortas from Wistar rats were excised, cleaned adhering tissue and cut into rings (5 mm). The rings were placed in 96-well plates containing DMEM and 300 µm L-NAME for 10 min. The plate was placed in a water bath (37 °C) for 5 min and then transferred back to a slush of ice. Aortic rings were rapidly transferred to Eppendorf tubes and immediately frozen in liquid nitrogen until analyzed by western blotting.

Aortic rings were manually cryogrinded using a small-sized mortar placed on liquid nitrogen. The pulverized tissue was brushed back into an Eppendorf tube placed in liquid nitrogen. The Eppendorf tube was subsequently placed on ice with the simultaneous addition of RIPA buffer containing protease inhibitor cocktail (Sigma Aldrich, P8340) and phosphatase inhibitor cocktail 2 (Sigma Aldrich, P5726) followed by immediate vortexing. The sample was thereafter placed on ice and vortexed additional two times every 10 min. Finally, the sample was sonicated for 3 s followed by centrifugation at 15,000*g* for 15 min, and the supernatant was transferred to an empty Eppendorf tube followed by protein quantification using a BCA protein assay (Micro BCA Protein Assay, Thermo Fisher Scientific). After adjusting protein concentration, samples were diluted in commercial 4x Laemmli sample buffer (Bio-Rad Laboratories) containing 10% 2*-*mercaptoethanol, then heated at 95 °C for 5 min and transferred to a freezer until analyzed.

Samples were separated by SDS-PAGE (Bio-Rad Laboratories; Criterion cell gradient gels, 4–20% acrylamide). Gels were equilibrated in a transfer buffer 20% methanol for ∼15 min before proteins were transferred to PVDF membranes. Membranes were blocked in TBST buffer (20 mM Tris base, 137 mM NaCl, 0.2% Tween-20, pH 7.6 and 5% nonfat dry milk) for 1 h at room temperature. Membranes were incubated with primary antibodies targeting phospho-VASP (Ser239; Cell Signaling Technologies, 3114) diluted 1:1,000 in TBST, 5% nonfat dry milk overnight. After washing, the membrane was incubated with secondary antibody, antirabbit IgG, HRP linked (Cell Signaling Technology, 7074) diluted 1:10,000 for 1.5 h at room temperature. Membranes were visualized by chemiluminescent detection using SuperSignal West Femto Chemiluminescent Substrate (Thermo Fisher Scientific). Bands were visualized using ChemiDoc MP (Bio-Rad Laboratories), and band intensities were analyzed by the software Image Lab 6.0.1 (Bio-Rad, Laboratories).

### Cell culture

HEK293A cells (LGC Standards) were grown in DMEM (high glucose) containing 10% FBS, 50 units per ml penicillin, 50 µg ml^−1^ streptomycin, 2 mM l-glutamine and 0.1 mM MEM nonessential amino acids at 37 °C. Cells were dissociated with trypsin and resuspended in a complete medium followed by centrifugation at 500*g* for 6 min. Pellets of cells were resuspended in PBS and counted by an automated cell counter (Bio-Rad, TC20).

### In vivo blood pressure recordings

Male Wistar rats received either L-NAME (1 g l^−1^) or placebo (NaCl) in the drinking water for 2 d before the cardiovascular recordings. We anesthetized the rats with isoflurane and implanted polyethylene catheters into the abdominal aorta and inferior vena cava through the femoral artery and vein for arterial pressure recordings and drug injections, respectively. We monitored mean blood pressure and heart rate in anesthetized animals using a pressure transducer coupled to an acquisition system (ADInstruments; PowerLab 8/35) connected to a computer running LabChart 7.0 software (ADInstruments). Cardiovascular responses to different doses of NO, oxidized Mb(Fe^3+^), dithionite-reduced Mb(Fe^2+^), NO-ferroheme-Mb or NO-ferroheme-l-Cys, administered intravenously, were evaluated after at least 30 min of stabilization. Special care was undertaken to protect solutions of NO, NO-ferroheme-Mb and NO-ferroheme-l-Cys from exposure to oxygen and light.

### Statistics and data presentation

Data were analyzed using GraphPad Prism software (v.9.5.0, (525)). Individual vessel concentration–response curves ex vivo and blood pressure responses in vivo were analyzed with two-way ANOVA with repeated measurements and followed by Šídák’s multiple comparisons test. For multiple comparisons of other parameters, a one-way ANOVA adjusted with Šídák’s correction was used. For comparisons between two treatments or two groups, data were analyzed with paired and unpaired two-sided Student’s *t*-test, respectively. Western blot data were analyzed by nonparametric Kruskal–Wallis test followed by Dunn’s multiple comparison test. Statistical significance was defined as *P* < 0.05. Data are expressed as mean ± s.d. unless otherwise indicated. *P ≤ 0.05, **P ≤ 0.01, ***P ≤ 0.001 and ****P ≤ 0.0001. Differences not significant are indicated as not significant (NS).

### Reporting summary

Further information on research design is available in the Nature Portfolio Reporting Summary linked to this article.

## Online content

Any methods, additional references, Nature Portfolio reporting summaries, source data, extended data, supplementary information, acknowledgements, peer review information; details of author contributions and competing interests; and statements of data and code availability are available at 10.1038/s41589-023-01411-5.

### Supplementary information

Supplementary Information Supplementary Figs. 1–8.

Reporting summary

Supplementary Data 1 Supporting data for Supplementary Fig. 1. Original data.

Supplementary Data 2 Supporting data for Supplementary Fig. 2. Original data.

Supplementary Data 3 Supporting data for Supplementary Fig. 7. Original data.

Supplementary Data 4 Supporting data for Supplementary Fig. 8. Original data.

### Source data

Source Data Figs. 1–6 Original data for figure graphs.

## Data Availability

The data supporting the findings of this study are available within the paper and its Supplementary Information. Source data are provided with this paper. Additional information is available from the authors upon request.
