# Supplementary material for: NO-ferroheme is a signaling entity in the vasculature
Source: Nat Chem Biol. 2023 Sep 14;19(10):1267–75. doi: 10.1038/s41589-023-01411-5 (PMC10522487; doi:10.1038/s41589-023-01411-5)
Supplement: Supplementary file 1 — Supplementary Figs. 1–8. [file 41589_2023_1411_MOESM1_ESM.pdf]

nature chemical biology

Article https://doi.org/10.1038/s41589-023-01411-5

NO-ferroheme is a signaling entity in the vasculature

In the format provided by the authors and unedited

Table of Contents

Supplementary Figures……………………………………………………………………………………………………….**1**

Supplementary Figure 1. Comparison of vasorelaxations induced by NO-ferroheme myoglobin and L-cysteine preparations……………………………………………………………………………………………………….1

Supplementary Figure 2. Vasorelaxation responses to NO-ferroheme myoglobin and authentic NO in intact and denuded vessels. ………………………………………………………………………………………………..2

Supplementary Figure 3. Partition of NO-ferroheme from NO-ferroheme-L-Cys solution to 1-octanol………3

Supplementary Figure 4. Partition of NO-ferroheme species to HEK 293 cells……………………………….4

Supplementary Figure 5. Partition of NO-ferroheme to RBC after in vivo administration of NO-ferroheme-Mb………5

Supplementary Figure 6. The cardiovascular effects *in vivo* of myoglobin (Mb), NO-ferroheme-Mb and NO preparations………………………………………………………………………………………………………6

Supplementary Figure 7. The cardiovascular effects *in vivo* of globin free preparation with NO-ferroheme-L-cysteine…………………………………………………………………………………………………….7

Supplementary Figure 8. Integral analysis of the cardiovascular responses to NO and NO-ferroheme preparations………8

Supplementary Figures

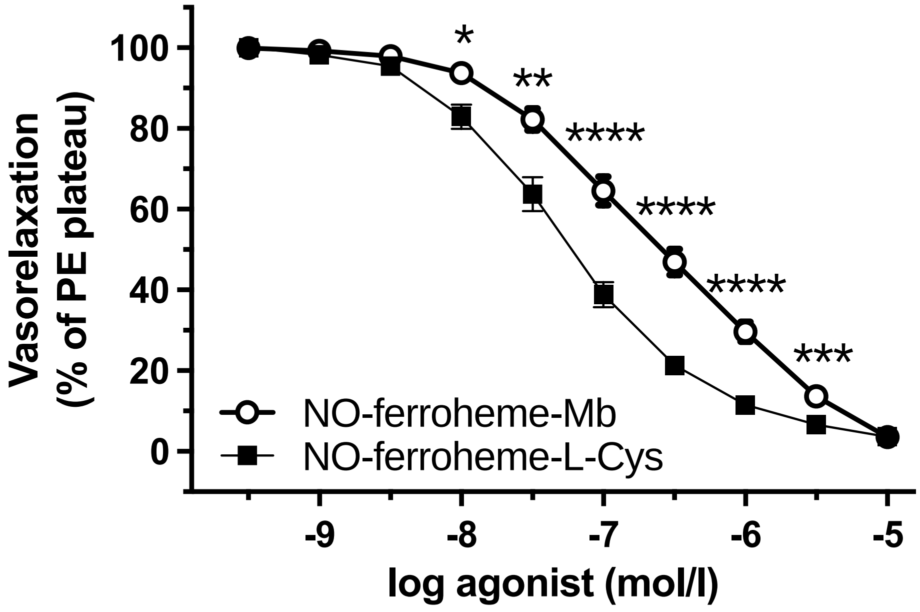

Supplementary Figure 1. Comparison of vasorelaxations induced by NO-ferroheme myoglobin and L-cysteine preparations.

Ex vivo vasorelaxation responses of aortic rings to increasing concentrations of NO-ferroheme-Mb *(n=31)* and NO-ferroheme-L-cysteine *(n=27)*, using the myograph system in the presence of 0.3 mM L-NAME. Data was analyzed by two-way repeated measures ANOVA followed by Šídák's multiple comparisons test. Vasorelaxation responses are shown as percent (%) of phenylephrine (PE)-induced plateau, and the data are presented as mean ± s.e.m. *n* represents the number of mouse aortic rings used in different myograph chambers. * denotes p ≤ 0.05, **p ≤ 0.01, ***p ≤ 0.001 and ****p ≤ 0.0001 respectively.

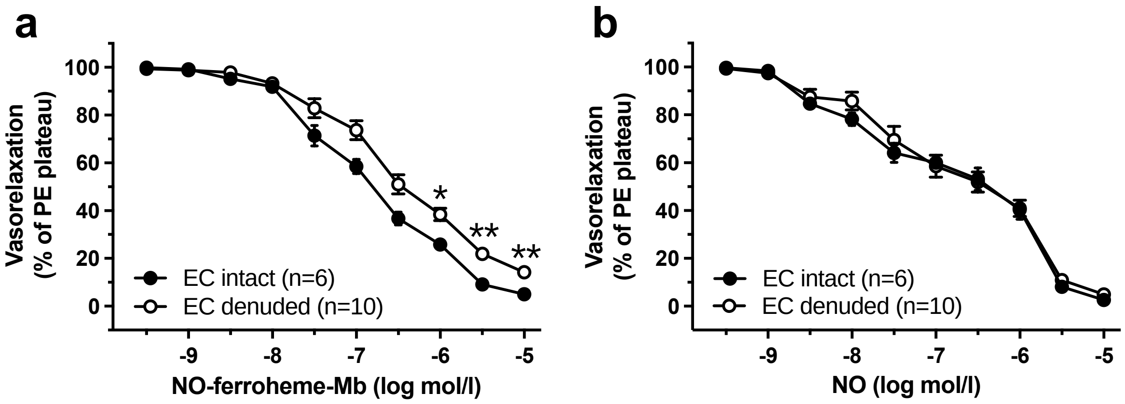

Supplementary Figure 2. Vasorelaxation responses to NO-ferroheme myoglobin and authentic NO in intact and denuded vessels.

Ex vivo vasorelaxation responses of aortic rings to increasing concentrations of NO-ferroheme-Mb (**a-b**) and authentic NO (**c-d**) in intact and denuded vessels with simultaneous treatment with the NOS inhibitor L-NAME (0.3 mmol/L). Data was analyzed by two-way repeated measures ANOVA followed by Šídák's multiple comparisons test. Vasorelaxation responses are shown as percent (%) of phenylephrine (PE)-induced plateau, and the data are presented as mean ± s.e.m. *n* represents the number of mouse aortic rings used in different myograph chambers. * denotes $p \leq 0.05$, **$p \leq 0.01$, respectively.

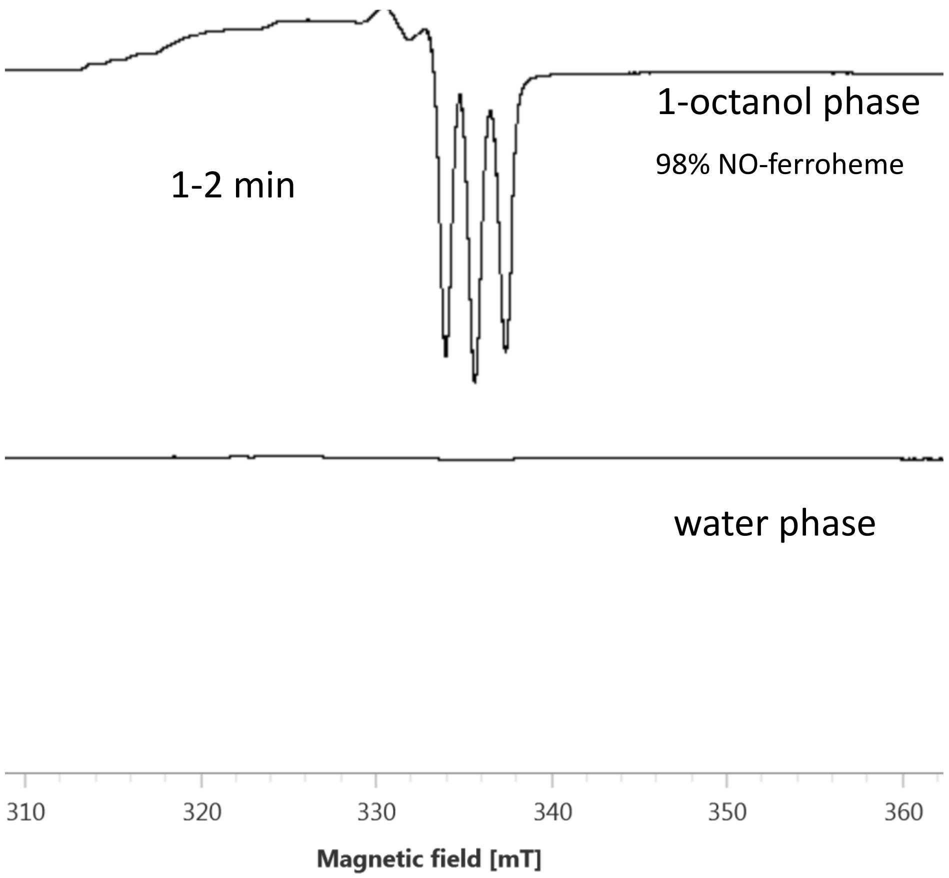

Supplementary Figure 3. Partition of NO-ferroheme from NO-ferroheme-L-Cys solution to 1-octanol.

Partition of NO-ferroheme as 5-coordinated species from NO-ferroheme-L-Cys preparation to 1-octanol phase. NO-ferroheme-L-Cys solution (0.5mM; 1 ml) was mixed with equal volume of deoxygenated 1-octanol, vortexed for 1min and centrifugated (2min). Upper (1-octanol) phase rapidly became red, while lower (water) phase became collarless. Samples from 1-octanol and water phases were analyzed by an X-band EPR at 77K. Double integration of the EPR signals indicated that 5-cood NO-ferroheme groups rapidly and nearly completely partitioned to 1-octanol phase. Representative spectra of 2 experiments.

a HEK cells + NO-ferroheme-Mb

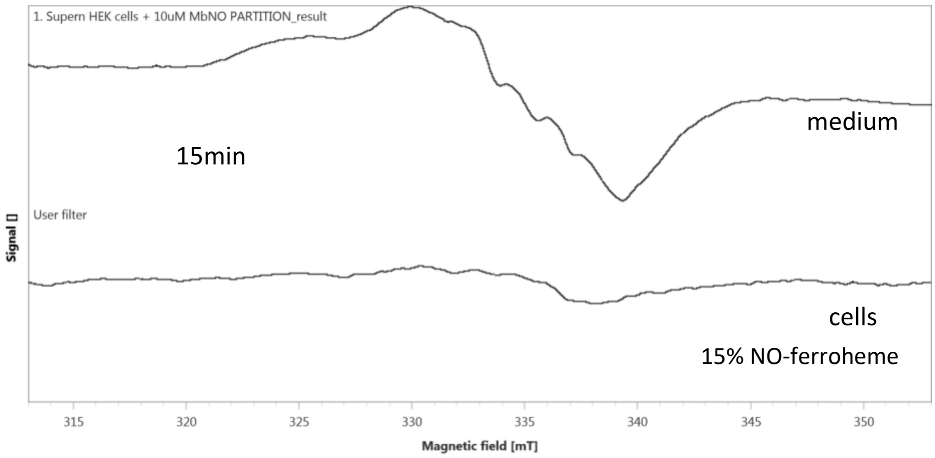

b HEK cells + NO-ferroheme-L-Cys

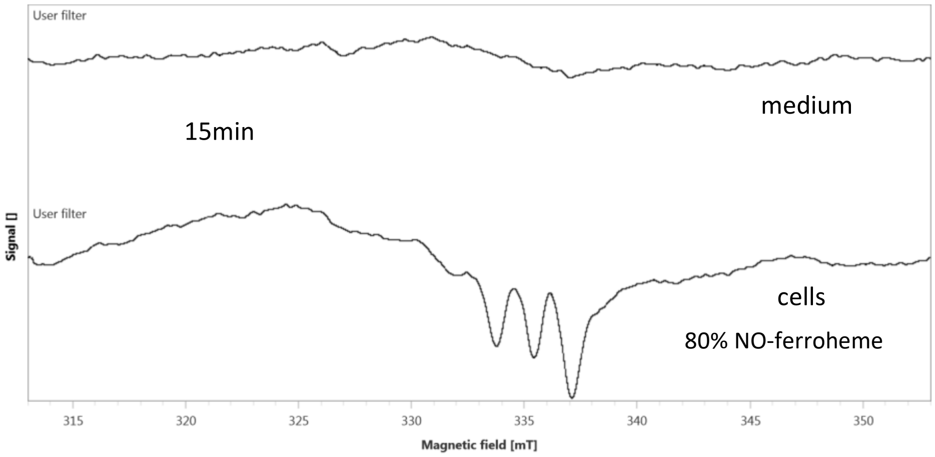

Supplementary Figure 4. Partition of NO-ferroheme species to HEK 293 cells.

Cell suspension (1:1 in PBS; $1.5x10^8$ cells/ml) were incubated (37°C; 15 min) either with 10 µM NO-ferroheme-Mb **(b)** or 10 µM NO-ferroheme-L-Cys **(b)** and then centrifugated. Samples from the medium/cells were analysed by an X-band EPR at 77K. Double integration of the EPR signals revealed that when NO-ferroheme-Mb was applied, about 15% of NO-ferroheme groups became associated with the cell fraction **(a)**; when NO-ferroheme-L-Cys were applied, up to 80% of NO-ferroheme groups were transferred to cells. Representative spectra of 2-3 experiments.

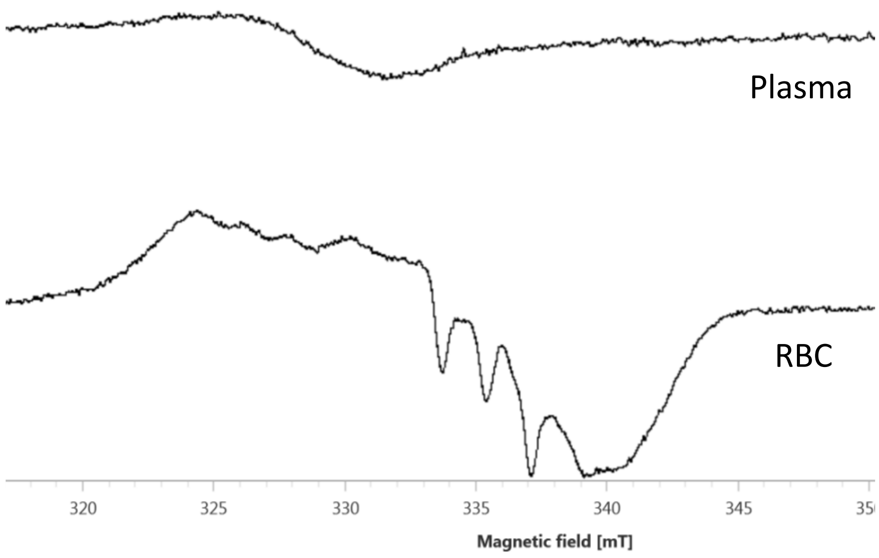

Supplementary Figure 5. Partition of NO-ferroheme to RBC after in vivo administration of NO-ferroheme-Mb.

Accumulation of NO-ferroheme species to red blood cells (RBC) 30 min after i.v. injection of NO-ferroheme-Mb (1600 nmol/kg). Blood plasma samples exhibited only EPR signal with g = 2.05, which is characteristic for Cu^{2+} ceruloplasmin. In contrast, RBCs demonstrated a mixed NO-ferroheme EPR signal. These data are consistent with our other results on NO-ferroheme transfer from exogenous NO-ferroheme-Mb to cells and tissues. EPR recording using an X-band EPR Magnettech 5000 at 77K.

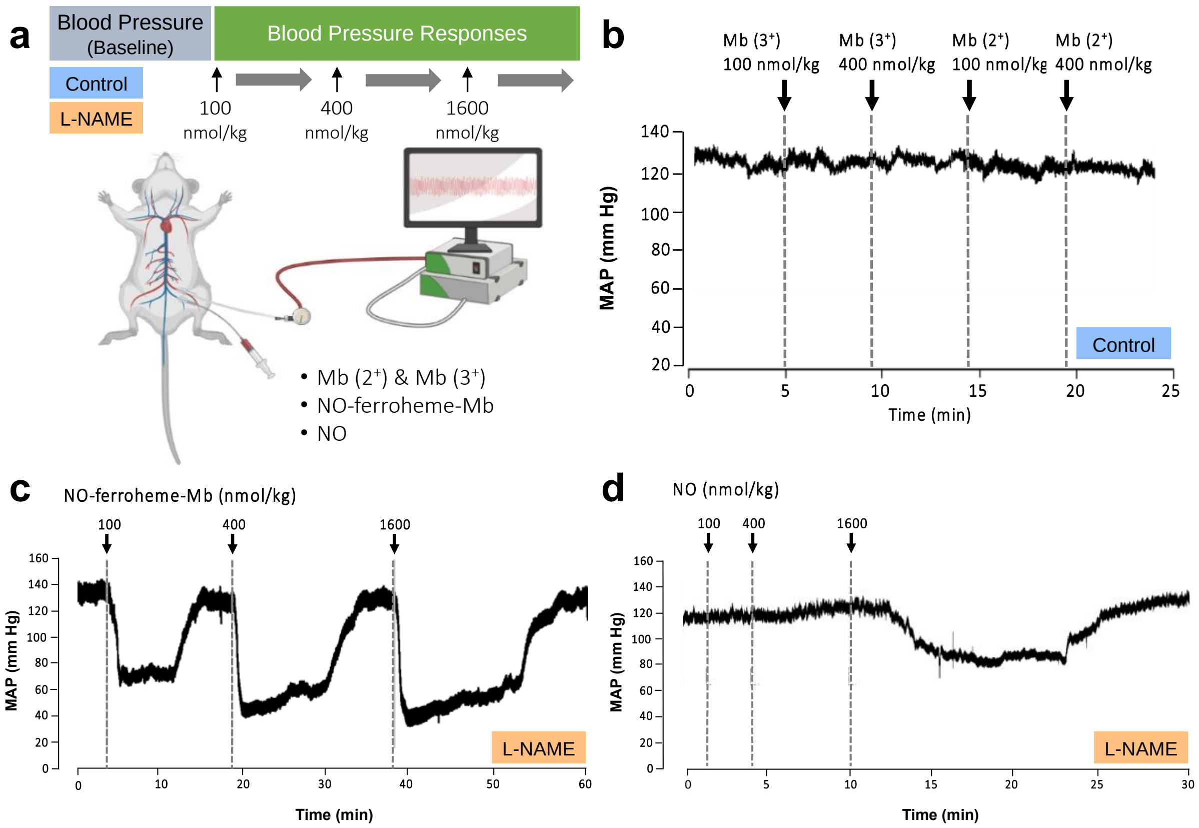

Supplementary Figure 6. The cardiovascular effects *in vivo* of myoglobin (Mb), NO-ferroheme-Mb and NO preparations.

a. The blood responses to intravenous injection of different doses of ferro-Mb (Mb^{2+}), ferri-Mb (Mb^{3+}), NO-ferroheme-Mb and authentic NO in anaesthetized Wistar rats with regular water or supplemented with L-NAME (1g/L). **b.** No significant effects of Mb(2^+) or Mb(3^+) on blood pressure were observed in control rats, as illustrated by representative trace. **c-d.** Traces of blood pressure in response to increasing bolus doses of NO-ferroheme-Mb (**c**) and authentic NO (**d**) in L-NAME treated rats. Administration of NO-ferroheme-Mb showed profound and dose-dependent reductions of blood pressure which were potentiated in rats with NOS inhibition. Authentic NO significantly reduced blood pressure only at highest dose (1600 nmol/kg) and this effect was not affected by L-NAME.

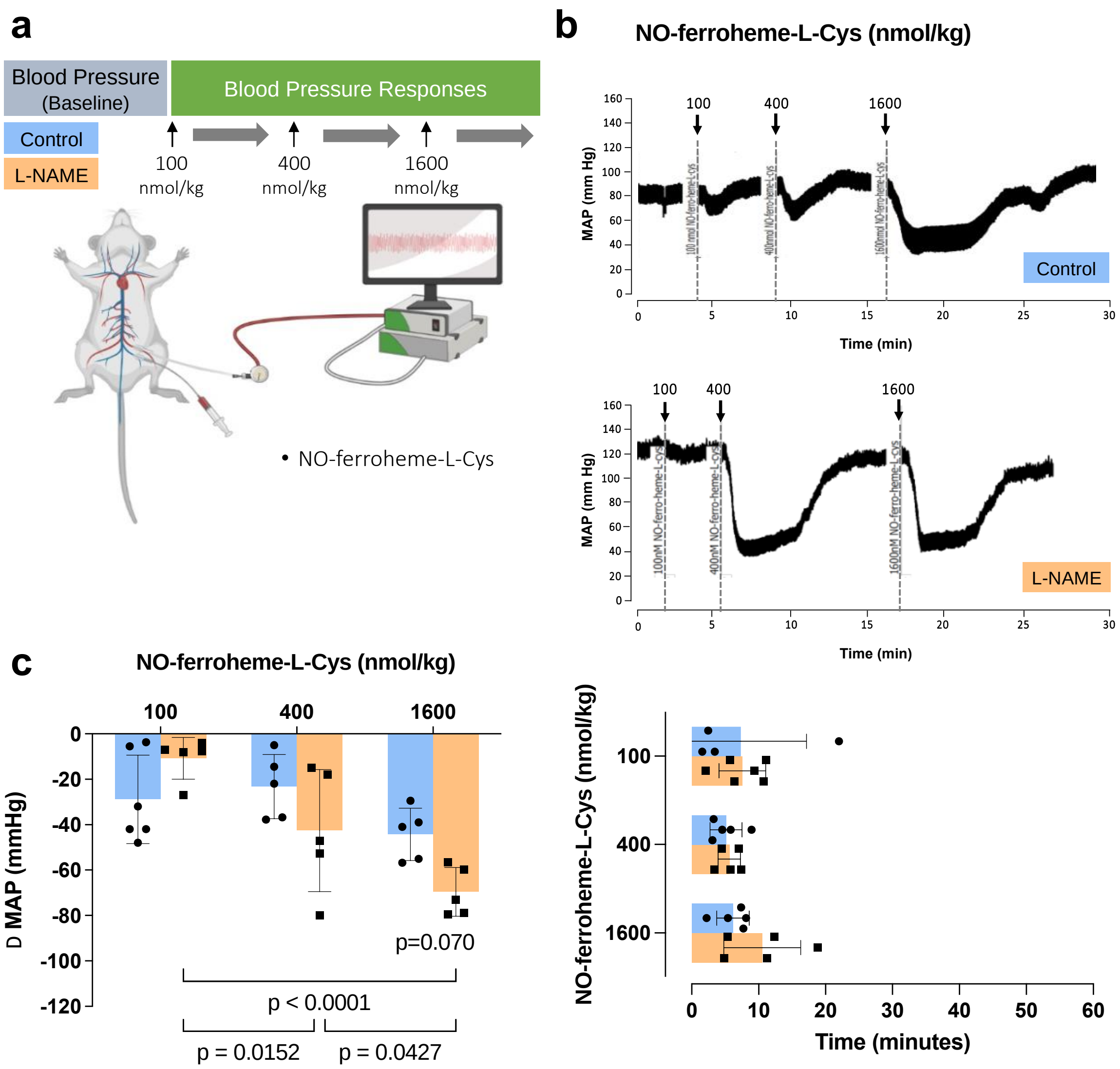

Supplementary Figure 7. The cardiovascular effects *in vivo* of globin free preparation with NO-ferroheme-L-cysteine.

a. The blood responses to intravenous injection of different doses of NO-ferroheme-L-Cys were analyzed in anaesthetized Wistar rats with regular water or supplemented with L-NAME (1g/L). **b.** Traces of blood pressure responses to increasing bolus doses of NO-ferroheme-L-cys in Control rats (*top panel*) and L-NAME treated rats (*lower panel*). **c.** Similar to NO-ferroheme-Mb, administration of NO-ferroheme-L-Cys dose-dependently lowered blood pressure and this effect was enhanced by L-NAME. However, the duration of the responses was shorter than that observed with NO-ferroheme-Mb (Compare with Figure 6 and Figure S3). Data analyzed with two-way ANOVA (mixed-effects model) followed by Šídák's multiple comparisons test. The dots in **c** represents the responses in different animals, and data are presented as mean ± s.d. P-values for statistically significant differences are indicated. Non-significant p-value (p=0.07) is comparison between Control and L-NAME treated rats given the highest dose.

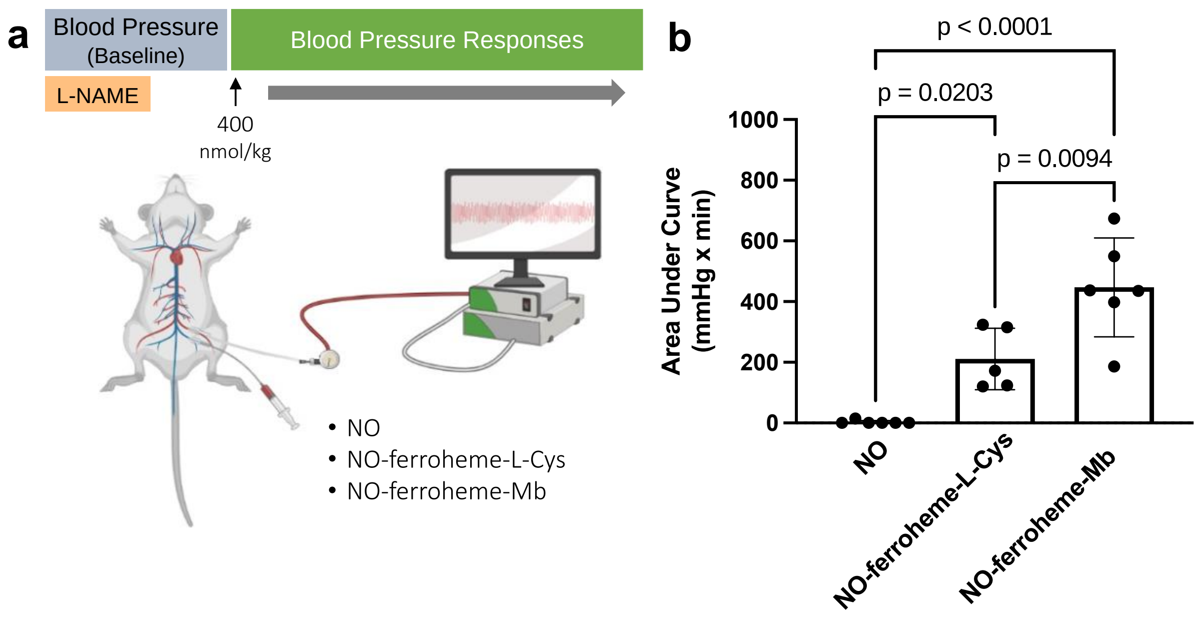

Supplementary Figure 8. Integral analysis of the cardiovascular responses to NO and NO-ferroheme preparations.

a. The overall blood responses to intravenous bolus injection of 400 nmol/kg of authentic NO, NO-ferroheme-L-Cys and NO-ferroheme-Mb were analyzed using LabChart reader v.8.1.22 and calculated as the integral of the total response (*i.e.* magnitude of blood pressure change in mmHg multiplied by the duration of the response in minutes). **b.** Analysis of the integral response (Area Under Curve) show that efficiency of NO-ferroheme-Mb was several-fold higher than that of NO-ferroheme-L-Cys, which in turn was more effective than authentic NO. Data analyzed with two-way ANOVA (mixed-effects model) followed by Šídák's multiple comparisons test. The dots in **b** represents the responses in different animals, and data are presented as mean ± s.d. P-values for statistically significant differences are indicated.
