# Supplementary material for: NO-ferroheme is a signaling entity in the vasculature
Source: Nat Chem Biol. 2023 Sep 14;19(10):1267–75. doi: 10.1038/s41589-023-01411-5 (PMC10522487; doi:10.1038/s41589-023-01411-5)
Supplement: Supplementary file 2 — Reporting summary [file 41589_2023_1411_MOESM2_ESM.pdf]

# nature portfolio

Corresponding author(s): Kleyschyov
Last updated by author(s): Jun 13, 2023

# Reporting Summary

Nature Portfolio wishes to improve the reproducibility of the work that we publish. This form provides structure for consistency and transparency in reporting. For further information on Nature Portfolio policies, see our Editorial Policies and the Editorial Policy Checklist.

## Statistics

For all statistical analyses, confirm that the following items are present in the figure legend, table legend, main text, or Methods section.

| n/a | Confirmed | |
|---|---|---|
| ☐ | ☒ | The exact sample size (*n*) for each experimental group/condition, given as a discrete number and unit of measurement |
| ☐ | ☒ | A statement on whether measurements were taken from distinct samples or whether the same sample was measured repeatedly |
| ☐ | ☒ | The statistical test(s) used AND whether they are one- or two-sided<br>*Only common tests should be described solely by name; describe more complex techniques in the Methods section.* |
| ☒ | ☐ | A description of all covariates tested |
| ☐ | ☒ | A description of any assumptions or corrections, such as tests of normality and adjustment for multiple comparisons |
| ☐ | ☒ | A full description of the statistical parameters including central tendency (e.g. means) or other basic estimates (e.g. regression coefficient) AND variation (e.g. standard deviation) or associated estimates of uncertainty (e.g. confidence intervals) |
| ☒ | ☐ | For null hypothesis testing, the test statistic (e.g. *F*, *t*, *r*) with confidence intervals, effect sizes, degrees of freedom and *P* value noted<br>*Give P values as exact values whenever suitable.* |
| ☒ | ☐ | For Bayesian analysis, information on the choice of priors and Markov chain Monte Carlo settings |
| ☒ | ☐ | For hierarchical and complex designs, identification of the appropriate level for tests and full reporting of outcomes |
| ☒ | ☐ | Estimates of effect sizes (e.g. Cohen's *d*, Pearson's *r*), indicating how they were calculated |

*Our web collection on statistics for biologists contains articles on many of the points above.*

## Software and code

Policy information about availability of computer code

| | |
|---|---|
| Data collection | Isometric tension was recorded with PowerLab system (PowerLab 4/30) and data collected with Labchart 7.0. Mitochondrial respiration was evaluated by high resolution respirometry (Oroboros, O2K) and data collected with Datlab 6.1.07.Mean blood pressure and heart rate were monitored in anesthetized animals using a pressure transducer coupled to an acquisition system (PowerLab; ADInstruments, Castle Hill, NSW, Australia) connected to a computer running LabChart 7.0 software (ADInstruments, Castle Hill, NSW, Australia). EPR spectra was measured with the EPR spectrometer MS5000 (Magnettech-Bruker, Freiberg, Germany). Gaseous NO was measured with ECO Physics chemiluminescense anlyser CLD 77AM and data collected with PowerChrom 2.1.9. Bands were visualized using ChemiDoc™ MP (Bio-Rad Laboratories) and band intensities analyzed by the software Image Lab 6.0.1 (Bio-Rad, Laboratories). |
| Data analysis | Graphpad Prism 9.5.0 was used to analyse statistics and create graphs |

For manuscripts utilizing custom algorithms or software that are central to the research but not yet described in published literature, software must be made available to editors and reviewers. We strongly encourage code deposition in a community repository (e.g. GitHub). See the Nature Portfolio guidelines for submitting code & software for further information.

## Data

Policy information about availability of data

All manuscripts must include a data availability statement. This statement should provide the following information, where applicable:

- Accession codes, unique identifiers, or web links for publicly available datasets
- A description of any restrictions on data availability
- For clinical datasets or third party data, please ensure that the statement adheres to our policy

The data supporting the findings of this study are available within the paper and its Supplementary Information. Source data are provided with this paper. Additional information is available from the authors upon reasonable request.

## Human research participants

Policy information about studies involving human research participants and Sex and Gender in Research.

| | |
|---|---|
| Reporting on sex and gender | N/A |
| Population characteristics | N/A |
| Recruitment | N/A |
| Ethics oversight | N/A |

Note that full information on the approval of the study protocol must also be provided in the manuscript.

# Field-specific reporting

Please select the one below that is the best fit for your research. If you are not sure, read the appropriate sections before making your selection.

☒ Life sciences ☐ Behavioural & social sciences ☐ Ecological, evolutionary & environmental sciences

For a reference copy of the document with all sections, see nature.com/documents/nr-reporting-summary-flat.pdf

# Life sciences study design

All studies must disclose on these points even when the disclosure is negative.

| | |
|---|---|
| Sample size | We have disclosed sample size. These were chosen based on our previous experiments in performing similar studies. In no case the sample size was lower than 4. For the in vivo experiments we used six animal in each group. Every experiment was preceeded by pilot experiments indicating the approximate sample size needed. |
| Data exclusions | No data have been excluded |
| Replication | We have indicated the number of times we have replicated the data in each experiment. All experiments were repeated at least 4 times and up to 31 times. In the vessel experiments aortas from at least different 4 animals were used. In the head space chemiluminescence experiments 5-7 independent measurements were performed in each group. In the blood pressure experiments 6 animals in each gropup were used. |
| Randomization | In the in vivo experiments the animals were randomized to receive either an NO-donor or NO-ferroheme and blood pressure was recorded by an un-blinded investigator. |
| Blinding | Investigators were not blinded in the study. Data (e.g., but not limited to, EPR spectra, mitochondrial respiration, blood pressure recordings and vessel relaxation) are not subjective measurements. Typically, the data collection software present a value or a progression of values that are registered and saved for further analysis. |

# Reporting for specific materials, systems and methods

We require information from authors about some types of materials, experimental systems and methods used in many studies. Here, indicate whether each material, system or method listed is relevant to your study. If you are not sure if a list item applies to your research, read the appropriate section before selecting a response.

## Materials & experimental systems

| n/a | Involved in the study |
|---|---|
| ☐ | ☒ Antibodies |
| ☐ | ☒ Eukaryotic cell lines |
| ☒ | ☐ Palaeontology and archaeology |
| ☐ | ☒ Animals and other organisms |
| ☒ | ☐ Clinical data |
| ☒ | ☐ Dual use research of concern |

## Methods

| n/a | Involved in the study |
|---|---|
| ☒ | ☐ ChIP-seq |
| ☒ | ☐ Flow cytometry |
| ☒ | ☐ MRI-based neuroimaging |

# Antibodies

| | |
|---|---|
| Antibodies used | Membranes were incubated with primary antibodies targeting Phospho-VASP (Ser239) (#3114, Cell Signaling Technologies) diluted 1:1000 in TBST, 5% nonfat dry milk overnight. After washing, membrane was incubated with secondary antibody, anti-rabbit IgG, HRP linked (#7074, Cell Signaling Technology) diluted 1:10000 for 1.5 h at room temperature. Membranes were visualized by chemiluminescent detection using SuperSignal West Femto Chemiluminescent Substrate (Thermo Scientific). Bands were visualized using ChemiDoc™ MP (Bio-Rad Laboratories) and band intensities analyzed by the software Image Lab 6.0.1 (Bio-Rad, Laboratories). |
| Validation | Cited in numerous publications e.g. PMID 34000062 |

# Eukaryotic cell lines

Policy information about cell lines and Sex and Gender in Research

| | |
|---|---|
| Cell line source(s) | HEK293A cells were obtained from LGC standards GmbH in Wesel Germany with the order code ATCC-CRL-2190. |
| Authentication | No method of cell line authentication was used |
| Mycoplasma contamination | The cell lines were not tested for mycoplasma. |
| Commonly misidentified lines (See ICLAC register) | No such cell lines were used in the study |

# Animals and other research organisms

Policy information about studies involving animals; ARRIVE guidelines recommended for reporting animal research, and Sex and Gender in Research

| | |
|---|---|
| Laboratory animals | Commercially available conventional male Wistar rats (200–250 g, 8-10 weeks old) and male C57BL/6 mice (20-25 g, 6-12 weeks pold) were purchased from Janvier Labs (France) and housed at the animal facility (KM-B) at the Karolinska Institutet. |
| Wild animals | No wild animals were used |
| Reporting on sex | Only male rodents were used. Variation in NO related enzymes are known to occur in the menstrual cycle. |
| Field-collected samples | No field collected samples |
| Ethics oversight | All experimental protocols were approved by the Stockholm Regional Institutional Animal Care and Use Committee (Dnr 17128-2021 and N139/15) and performed according to the US National Institutes of Health guidelines (NIH publication NO. 85-23, revised 1996) and EU directive 2010/63/EU for the conduct of experiments in animals. |

Note that full information on the approval of the study protocol must also be provided in the manuscript.
